# A Multigene Approach for Comparing Genealogy of *Betacoronavirus* from Cattle and Horses

**DOI:** 10.1155/2013/349702

**Published:** 2013-11-17

**Authors:** Iracema N. Barros, Sheila O. S. Silva, Francisco S. Nogueira Neto, Karen M. Asano, Sibele P. Souza, Leonardo J. Richtzenhain, Paulo E. Brandao

**Affiliations:** ^1^Department of Preventive Veterinary Medicine and Animal Health, College of Veterinary Medicine, University of São Paulo, Avenue Professor Dr. Orlando Marques de Paiva 87, Cidade Universitária, 05508-270 São Paulo, SP, Brazil; ^2^Coronavirus Research Group, College of Veterinary Medicine, University of São Paulo, Avenue Professor Dr. Orlando Marques de Paiva 87, Cidade Universitária, 05508-270 São Paulo, SP, Brazil; ^3^Jockey Club of São Paulo, Bento Frias Street 248, Group 555, 05423-050 São Paulo, SP, Brazil

## Abstract

Gastroenteritis is one of the leading causes of morbidity and mortality among young and newborn animals and is often caused by multiple intestinal infections, with rotavirus and bovine coronavirus (BCoV) being the main viral causes in cattle. Given that BCoV is better studied than equine coronaviruses and given the possibility of interspecies transmission of these viruses, this research was designed to compare the partial sequences of the spike glycoprotein (S), hemagglutinin-esterase protein (HE), and nucleoprotein (N) genes from coronaviruses from adult cattle with winter dysentery, calves with neonatal diarrhea, and horses. To achieve this, eleven fecal samples from dairy cows with winter dysentery, three from calves, and two from horses, all from Brazil, were analysed. It could be concluded that the enteric BCoV genealogy from newborn and adult cattle is directly associated with geographic distribution patterns, when S and HE genes are taken into account. A less-resolved genealogy exists for the HE and N genes in cattle, with a trend for an age-related segregation pattern. The coronavirus strains from horses revealed *Betacoronavirus* sequences indistinguishable from those found in cattle, a fact previously unknown.

## 1. Introduction

Currently, coronaviruses (CoVs) with genetic and antigenic proximities to bovine coronavirus (BCoV) such as human coronaviruses HCoV-OC43, porcine hemagglutinating encephalomyelitis virus (PHEV), and equine coronavirus (EqCoV) are not considered to be separate species but as belonging to the species *Betacoronavirus-1* within the genus *Betacoronavirus.* This genus has replaced Group 2 in the order Nidovirales, family Coronaviridae, and according to the new taxonomy; this family is separated into two subfamilies: Torovirinae and Coronavirinae. The latter comprises the genera *Betacoronavirus, Alphacoronavirus,* and *Gammacoronavirus* [1, 2]. 

CoVs are enveloped, single-stranded positive sense RNA viruses with a genome encoding replicase polyproteins, the four structural proteins: spike (S) glycoprotein (a receptor-interacting and a target for neutralizing antibody in the envelope); nucleocapsid (N) (associated with the genomic RNA in the nucleocapsid); and the two proteins essential for virion formation, envelope (E) and membrane (M) proteins; some *Betacoronaviruses *also present the hemagglutinin-esterase (HE) protein, with the role as a secondary receptor-binding envelope protein and accessory proteins [3, 4].

BCoV is a major pathogen for cattle, frequently found in neonatal diarrhea, dysentery in the adult and respiratory disease [5, 6]. Similarly, in horses, coronaviruses lead to neonatal enterocolitis [7, 8], although there are very few studies on the genealogy of coronaviruses from these animals [7, 9, 10].

Given the little information available on coronaviruses of horses and the genealogic relationship of these coronaviruses from cattle, this research was designed to perform a multigenic comparison of coronaviruses from adult cattle with winter dysentery, calves with neonatal diarrhea, and horses based on partial sequences of the HE, S, and N genes.

## 2. Materials and Methods

### 2.1. Controls

BCoV Kakegawa strain [11] grown in hamster lung (HmLu) cells, with a hemagglutination titer of 256 and DEPC-treated water, was used as positive and negative controls, respectively.

In the nested RT-PCRs, DEPC-treated water was used as a negative control every five samples, also added to the mix, and placed in a thermocycler in order to monitor contamination by DNA amplicons. Each step of the study (RNA extraction, nested RT-PCR, electrophoresis, and DNA sequencing) was carried out in different rooms with materials and reagents exclusive for that specific step in order to prevent DNA carryover. 

### 2.2. Field Samples

**Figure 1:**
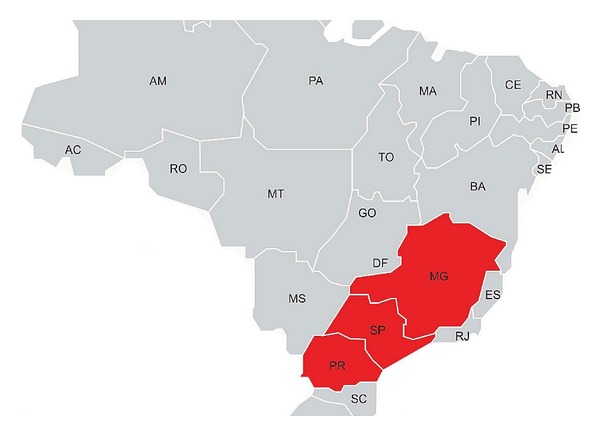
Map of Brazil highlighting SP, PR and MG states.

Fecal samples were collected from eight dysenteric and three healthy adult cows (named B1 to B11) in 2010 from a farm in Parana State, Southern Brazil; two samples came from healthy young adult horses (E17 and E19) in 2009 in a farm in São Paulo State, Southeastern Brazil and three fecal samples from dairy calves with neonatal diarrhea (USP01, USP03, and USP05) collected in the state of MG, Southeastern Brazil, in 2001. BCoV in these last three calf samples had previously been studied for S gene genealogy [12] (GenBank accession numbers AY255831, AY606193, and AY606195). These states are shown on the map (Figure 1). 

Samples were prepared as 20% suspensions in DEPC-treated water and centrifuged at 5,000 ×g/15 min at 4°C, and the supernatant was stored at −80°C prior to analysis.

### 2.3. Partial HE, S, and N genes Amplification

Total RNA was extracted from the supernatants with the TRIzol reagent (Invitrogen, Carlsbad, CA, USA), and cDNA was synthesized using random primers (Invitrogen, Carlsbad, CA, USA) and M-MLV reverse transcriptase (Invitrogen, Carlsbad, CA, USA) as described by the manufacturer.

Amplification of partial HE (nucleotides 122 to 562), S (nt. 1312 to 1799), and N (nt. 123 to 428) genes was performed as described by Souza et al. [13], Brandao et al. [12], and Asano et al. [14], using Platinum Taq DNA polymerase (Invitrogen, Carlsbad, CA, USA) according to the manufacturer's instructions. Nucleotide positions refer to the Mebus strain (GenBank accession number U00735.2).

### 2.4. DNA Sequencing and Genealogy

Amplicons for each gene (HE: 441bp; S: 488bp; and N: 306bp) were purified from agarose gels with the GFX PCR DNA and GB Purification Kit (GE Healthcare Bio-sciences Corp, Piscataway, NJ, USA) and submitted to bidirectional sequencing with BigDye version 3.1 (Applied Biosystems, Carlsbad, CA, USA) according to the manufacturer's instructions. Sequences were resolved in an ABI-377 sequencer.

Chromatograms were submitted to Phred analysis (http://asparagin.cenargen.embrapa.br/phph/), and positions with scores >20 were used to assemble sequences with CAP-Contig in BioEdit 7.0.9.0 [15].

The sequences were then aligned with BCoV and EqCoV homologous sequences retrieved from GenBank (accession numbers for HE gene: AF391541, EF424619, AF058944, EF424620, EF424615, EF424618, EF424616, GU214763, GU214757, GU214765, GU214769, GU214767, GU214766, GU214768, GU214761, AF058943, DQ811784, AF220295, AB354579, U00735, AF058942, AY585229, AY316300, NC010327, and EF446615; S gene AY606198, AY606205, AY606203, AY606202, AY606192, AY606204, AY606196, AY606197, AY606200, AY606194, AY606199, AY606201, DQ479424, U00735, AB354579, AF220295, EF424620, AF239307, AF391541, DQ811784, U06093, AF058942, EF424619, EF424615, AF058944, EF424616, EF424618, FJ899737, AF058943, DQ479423, DQ479421, DQ479422, AY585229, AY316300, EF446615, and NC_010327; and N gene AB354579, AF220295, U00735, AF058942, AY5852229, AF251144, NC010327, EF446615, AF058943, GU808349, GU808341, GU808345, GU808344, EF424619, GU808343, GU808348, EF424616, DQ811784, EF424618, EF424615, EF424620, GU808350, AF391541, and AF058944) using CLUSTAL/W with BioEdit 7.0.9.0 [15] and the BLOSSUM62 matrix (for the putative amino acids sequences). Distance phylogenetic trees were calculated using the Neighbor-Joining algorithm and Maximum Composite Likelihood models for nucleotides (nt.) sequences and the Poisson correction for amino acid (aa) sequences with 1,000 bootstrap replicates with MEGA 4 [16]. *Canine coronavirus* (CCoV, *Alphacoronavirus*) S and N genes and *Murine hepatitis virus* (MHV, *Betacoronavirus*) HE genes (absent in *Alphacoronavirus*) were used as outgroups. MHV was not used as outgroups for S and N in order to have an outgroup that diverged from the ingroup (*Betacoronavirus*) before the internal speciation of this group.

## 3. Results 

**Figure 2:**
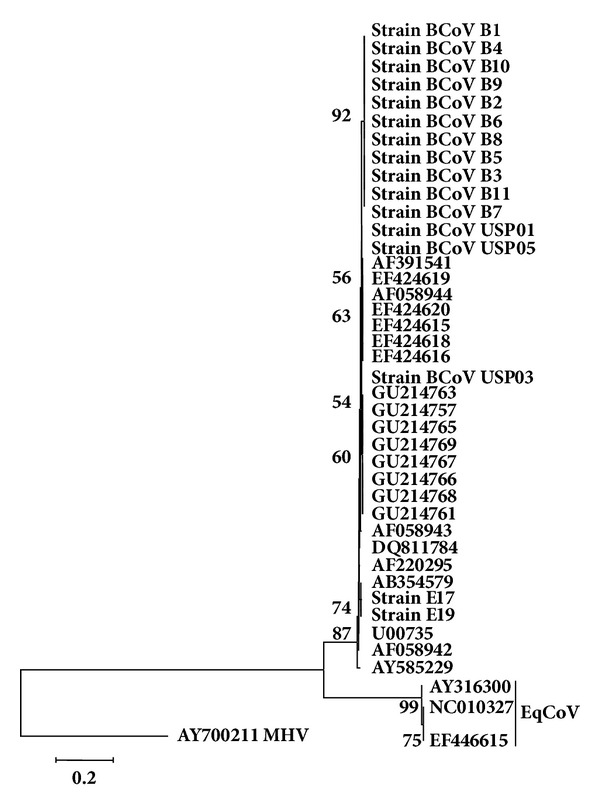
Phylogenetic tree for partial sequences of HE gene (nucleotides 189 to 521 of Mebus strain GenBank accession number U00735.2). Strains of the present study are in bold. The numbers at each node are the bootstrap values obtained with 1,000 replicates (only values >50 are shown). The bar represents the number of substitutions per site.

The tree based on *HE* nt. sequences (Figure 2) showed that both equine strains which segregated with BCoV strains from this study and others retrieved from GenBank, are included in the same subcluster of BCoV Kakegawa strain (GenBank accession number AB354579), divergent from EqCoV strains (AY316300, NC_010327, and EF446615). 

In this tree, the BCoV strains found in adult dairy cows and calves analyzed in this study segregated in a single cluster, together with other BCoV strains. 

Regarding the nt. and aa for the *HE *sequences, the identity amongst the two equine strains E17 and E19 and groups of BCoV strains ranged from 97.35 to 98.25% and 96.81 to 97.65%, respectively. However, amongst these two equine strains and EqCoV strain, the lowest identity percentage was 71.65% for aa and the highest was 72.85% for nt. Besides, the identity amongst groups of BCoV strains studied herein and other BCoV strains ranged from 98.19 to 99.60% and 98.85 to 100% for nt. and aa, respectively. 

**Figure 3:**
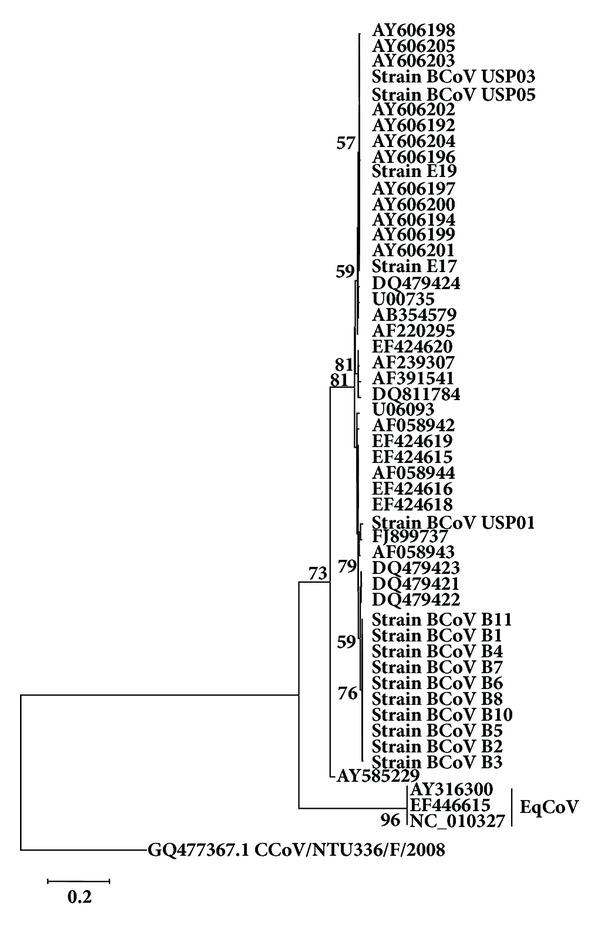
Phylogenetic tree for partial sequences of S gene (nt. 1395 to 1667 of Mebus strain GenBank accession number U00735.2). Strains of the present study are in bold. The numbers at each node are the bootstrap values obtained with 1,000 replicates (only values >50 are shown). The bar represents the number of substitutions per site.

Taking into account the nt. sequence tree for the S gene (Figure 3), equine strains E17 and E19 segregated amongst BCoV strains from calves of Southeastern region of Brazil, identified as USP and again diverged from EqCoV strains (AY316300, NC_010327, and EF446615).

The cluster with these two equine strains also contains BCoV strains described by Brandão et al. [12] in which a deletion of 18 nt./6aa in the S1 subunit region of S protein was detected, also found in E17 and E19 strains. In turn, strain USP01, which did not have the abovementioned deletion, segregated with the Brazilian strain cow/WDBR-96/BRA/2003 (FJ899737), also from Southeastern region of Brazil. BCoV strains from adult dairy cows segregated in a single cluster, as described for HE gene. 

Considering the nt. and aa identities for S gene, the lowest identity found amongst the two equine studied strains and groups of BCoV was 89.27% or 89.60% and the highest identities were 99.92% and 99.83%, respectively. Amongst these two equine strains and EqCoV strains, the lowest and highest identities were 47.2% (nt.) and 56.8% (aa), and the identity amongst groups of BCoV strains studied herein and other BCoV strains ranged from 89.20 to 98.17% and 86.45 to 97.89% for nt. and aa, respectively. 

**Figure 4:**
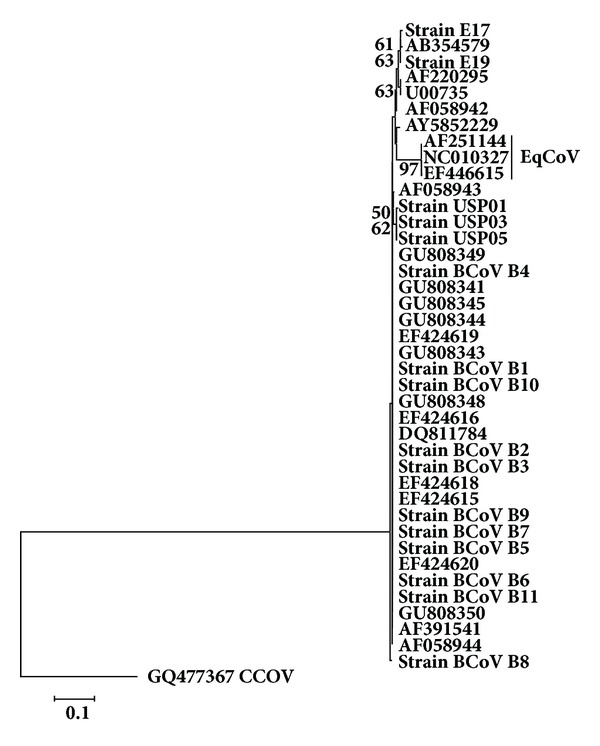
Phylogenetic tree for partial sequences of N gene (nt. 156 to 359 of Mebus strain GenBank accession number U00735.2). Strains of the present study are in bold. The numbers at each node are the bootstrap values obtained with 1,000 replicates (only values >50 are shown). The bar represents the number of substitutions per site.

The tree for N gene (Figure 4) showed that both equine strains segregated in the same cluster of Kakegawa BcoV (AB354579), similar to that found in the nt. sequences tree for HE gene and also diverged from EqCoV strains (AY316300, NC_010327, and EF446615). The BCoV strains from calves from Southeastern Brazil segregated in a distinct subcluster to the BCoV strains of adult cows, similar to that found for genes HE and S, although a lower resolution was found in the N gene tree, since the subclusters described for S and HE genes were not detected, without geographical distinction. 

Regarding the identities of nucleotides and amino acids for the N gene, the lowest identities found amongst the equine strains E17 and E19 and BCoV strains groups were 96.94% and 97.18% and the highest was 97.73% or 97.5%, respectively. Amongst these two strains and EqCoV strains, the lowest and highest percentage identities were 92% (nt.) and 95.10% (aa). The E17 strain showed a G274C nucleotide substitution, resulting in a Val92Leu change, thereby distinguishing this strain from the E19 and other BCoV strains. However, identity amongst groups of BCoV strains studied herein and published BCoV strains ranged from 98.62 to 99.89% and 99.29 to 100% for nt. and aa, respectively. 

The nt. sequence of all genes studied in this study has been deposited in the GenBank under accession numbers (Table 1).

## 4. Discussion and Conclusions

With respect to the genealogical analysis, the coronavirus strains E17 and E19, detected in horses, did not cluster with EqCoV strains already described [7, 9]. Nonetheless the genes analysed, unexpectedly, clustered with BCoV strains. These results demonstrate that the coronaviruses found in these horses are divergent from EqCoV and similar to BCoV.

The presence of coronaviruses similar to BCoV in hosts other than cattle has already been reported in buffalos [17, 18], lamas and alpacas [19, 20], deer [21], and giraffes [22], demonstrating that this virus can adapt to other herbivores, including horses, as found in the present study, a fact not reported previously. 

Furthermore, the 18 nt./ six aa deletion in S1, already described for BCoV strains [12], was also detected in strains E17 and E19, possibly allowing for changes in the spike glycoprotein that could reduce crossed immunity with other BCoV strains [23].

Regarding the HE gene, strains E17 and E19 both clustered with Kakegawa BCoV strain (Figure 2; Genbank ID: AB354579). Though this BCoV strain originated in Japan [11], it is possible that a common ancestor of the three strains has spread worldwide. 

Alternatively, BCoV strains from bovine resulted in three main clusters for *HE*: (a) strains from adult cows studied herein; (b) strains from dairy cows with winter dysentery previously reported in Brazil (Genbank); and (c) strains from cattle from other countries retrieved from the GenBank. 

This model of segregation might represent a phylogeographic pattern rather than temporal and/ or host-specific patterns, since it is known that there are no markers to differentiate strains of BCoV from calves and adult cattle [24, 25] and either for temporal changes [26–28]. A similar pattern of segregation for *HE *was maintained for *S *(Figure 3), strengthening the hypothesis of regional genic signatures. 

Considering the N gene tree (Figure 4), the strain E17 has diverged from E19 and BCoV strains, owing to a single nucleotide substitution, leading to aa substitution Val92Leu, increasing, for this strain, the number of nonsynonymous substitutions, which might mean that strain E17 is adapting to the equine host, and increasing its divergence from an ancestor BCoV strain. 

Thus, one can speculate that successive natural passages of the strain E17 amongst horses, without the participation of cattle, led to different host-parasite relationships due to differences in receptors, in viral replication, and in the intracytoplasmic content [29], and probably, this distance has a tendency to rise over evolutionary time. 

In the nucleotide tree for the N gene, two clusters of BCoV strains were formed; one cluster containing strains from calves (USP) and another with all of the remaining strains. Considering that *N* is the most conserved gene amongst those studied herein [4, 30], one can speculate that the strains from calves in this case have markers for the discrimination amongst strains from neonatal diarrhea and winter dysentery in cattle, in that the clustering was maintained despite the lack of geographical differentiation.

The debate on the taxonomy of coronaviruses has firstly led to the proposition of 3 groups in the genus *Coronavirus* [30] and became quite controversial after the description of the SARS coronavirus [31], in which taxonomy culminated with the proposition of a fourth group [32] which was then refuted and the virus was finally classified as a Group 2 member [33]. The newly proposed taxonomy for the Nidovirales, with the three coronavirus genera replacing the three groups, represents a great advance in organizing the increasing number of coronavirus “species” constantly being discovered.

In conclusion, the genealogy of enteric BCoVs from newborn and adult cattle is directly associated with geographical patterns, when the S and HE genes are taken into account, with a less-resolved genealogy for the HE and N genes, and with a trend for an age-related segregation pattern for the last, and horses might present *Betacoronavirus* highly similar to those found in cattle, supporting the existence of the *Betacoronavirus-1* species.

## Figures and Tables

**Table 1:** GenBank accession numbers of the strains studied; (—) not shown.

Strain/gene	HE	S	N
E17	JF345127	JF345143	JF345155
E19	JF345128	JF345144	JF345156
B1	JF345129	JF345145	JF345157
B2	JF345130	JF345146	JF345158
B3	JF345131	JF345147	JF345159
B4	JF345132	JF345148	JF345160
B5	JF345133	JF345149	JF345161
B6	JF345134	JF345150	JF345162
B7	JF345135	JF345151	JF345163
B8	JF345136	JF345152	JF345164
B9	JF345137	—	JF345165
B10	JF345138	JF345153	JF345166
B11	JF345139	JF345154	JF345167
USP01	JF345140	AY255831 (Brandao et al. 2006 [12])	JF345168
USP03	JF345141	AY606193 (Brandao et al. 2006 [12])	JF345169
USP05	JF345142	AY606195 (Brandao et al. 2006 [12])	JF345170
